# Sleep Disturbances and Emotional and Behavioral Difficulties Among Preschool-Aged Children

**DOI:** 10.1001/jamanetworkopen.2023.47623

**Published:** 2023-12-14

**Authors:** Yujiao Deng, Zichen Zhang, Yiding Gui, Wen Li, Tingyu Rong, Yanrui Jiang, Qi Zhu, Jin Zhao, Yunting Zhang, Guanghai Wang, Fan Jiang

**Affiliations:** 1 Department of Neurology, Shanghai Children’s Medical Center, School of Medicine, Shanghai Jiao Tong University, Shanghai, China; 2 Department of Developmental and Behavioral Pediatrics, Shanghai Children’s Medical Center, School of Medicine, Shanghai Jiao Tong University, Shanghai, China; 3 Pediatric Translational Medicine Institute, Shanghai Children’s Medical Center, School of Medicine, Shanghai Jiao Tong University, Shanghai, China; 4 Ministry of Education–Shanghai Key Laboratory of Children’s Environmental Health, Xinhua Hospital, School of Medicine, Shanghai Jiao Tong University, Shanghai, China; 5 Department of Pediatrics, The First Affiliated Hospital of Zhengzhou University, Zhengzhou, China; 6 Department of Child and Adolescent Healthcare, Children’s Hospital of Soochow University, Suzhou, China; 7 Child Health Advocacy Institute, Shanghai Children’s Medical Center, School of Medicine, Shanghai Jiao Tong University, Shanghai, China; 8 Shanghai Center for Brain Science and Brain-Inspired Technology, Shanghai, China

## Abstract

**Importance:**

Sleep disturbances and mental health problems are highly comorbid and bidirectionally correlated across childhood. The association between the natural history of sleep disturbances and the transition of mental health problems has not been quantified.

**Objective:**

To examine the association between the natural history of sleep disturbances and resolved and incident emotional and behavioral difficulties (EBDs).

**Design, Setting, and Participants:**

This cohort study used data from the Shanghai Children’s Health, Education and Lifestyle Evaluation–Preschool (SCHEDULE-P), a prospective and population-based longitudinal cohort study of children enrolled in preschools in Shanghai, China, from November 10 to 24, 2016. A total of 20 324 children aged 3 to 4 years were recruited from the junior class of 191 kindergartens, of whom 17 233 (84.8%) participated in the 2-year follow-up. A multilevel regression model was used to evaluate the association between the development of sleep disturbances and the occurrence of resolved and incident EBDs. The data analysis spanned from August 4, 2021, to October 31, 2023.

**Exposures:**

Sleep disturbances were assessed using the Children’s Sleep Habit Questionnaire; EBDs were assessed using the Strengths and Difficulties Questionnaire.

**Main Outcomes and Measures:**

Occurrence of incident and resolved EBDs at the 2-year follow-up.

**Results:**

The cohort included 17 182 participants, with a mean (SD) age of 3.73 (0.29) years at enrollment; 52.0% were boys. The prevalence of EBDs at school entry and graduation years was 27.8% and 18.7%, respectively, while the prevalence of sleep disturbances was 41.3% and 31.5%, respectively. Among those with EBDs at the entry year, 35.0% maintained stability in the graduation year, while sleep disturbances were stable in 50.0% of those with sleep disturbances. After controlling for confounding factors, the odds ratio (OR) for resolved EBDs was lower in the incident sleep disturbance (ISD) group (OR, 0.50 [95% CI, 0.41-0.62]; *P* < .001) and stable sleep disturbance (SSD) group (OR, 0.47 [95% CI, 0.40-0.56]; *P* < .001) compared with the group with no sleep disturbances. The ORs for incident EBDs among the ISD group (OR, 2.58 [95% CI, 2.22-3.01]; *P* < .001) and SSD group (OR, 2.29, [95% CI, 1.98-2.64]; *P* < .001) were higher than among the group with no sleep disturbances.

**Conclusions and Relevance:**

In this prospective cohort study, the natural history of sleep disturbances among preschool-aged children was associated with both resolved and incident EBDs. Routine screening and precise intervention for sleep disturbances may benefit the psychosocial well-being of this population.

## Key Points

**Question:**

Is a natural history of sleep disturbances associated with resolved and incident emotional and behavioral difficulties (EBDs) during preschool years?

**Findings:**

In this cohort study of 17 182 preschool-aged children in Shanghai, China, incident and stable sleep disturbances were negatively associated with resolved EBDs but positively associated with incident EBDs after adjustment. Resolved sleep disturbances were positively associated with resolved EBDs.

**Meaning:**

These findings underscore the association between sleep disturbances and EBDs among preschool-aged children and the importance of sleep health in promoting their mental well-being.

## Teaser

This cohort study examines how the natural history of sleep disturbances is associated with resolved and incident emotional and behavioral difficulties among preschool-aged children in Shanghai, China.

## Introduction

Mental health problems are a leading cause of the global burden of disease in children and adolescents.^1^ Psychological disorders may have an early onset in preschool years,^2^ with documented changes in brain function^3^ and structure^4^ involved in emotion processing. Emotional and behavioral difficulties (EBDs) among preschool-aged children deserve particular attention due to their high prevalence and detrimental effects on optimal mental health and functioning throughout childhood and into later life.^5,6,7^ Although EBDs may resolve as the child grows up, 49.9% of young children still exhibit persistence 1 year after initial presentation.^8^ Furthermore, while early intervention in a clinical setting can improve childhood EBDs,^9,10,11^ multiple barriers such as high training demands, stigma, availability, and cost of services prevent children from receiving treatment.^12,13^ These data highlight the need to identify both protective and risk factors that are modifiable in large community samples to prioritize more effective and scalable prevention and intervention strategies for EBDs in early childhood.

Emerging evidence indicates that poor sleep is one of the most important precursors of and risk factors for children’s EBDs.^14,15,16^ Children with preschool-onset sleep disturbances^17,18^ are reported to experience more internalizing and externalizing problems. For preschool-aged children, parent training intervention is considered a cost-effective strategy for managing their sleep disturbances,^11^ but its subsequent benefits on their social and emotional well-being are limited.^19^ On the other hand, little evidence has determined whether the changes in mental health are specifically due to sleep improvement.^13^ A clinical observational cohort study has the unique advantage of providing a more ecological inspection of the association between the natural transition of sleep disturbances and resolution of EBDs, which would extend global interest in planning low-threshold early sleep interventions and services for children with EBDs.

Most of the existing research tends to focus on general sleep disturbances, leaving unclear which specific type is most likely to be associated with EBDs. Several behavioral sleep disturbances (eg, sleep anxiety, night waking, insufficient sleep, and insomnia) have been associated with concurrent internalizing problems^20^ and later anxiety or depressive symptoms in children,^21^ whereas parasomnias^22,23^ or sleep-disordered breathing (SDB)^24^ have been linked to increased externalizing problems. In addition, studies have shown more temperamental difficulties in children with SDB than in their counterparts with behavioral insomnia.^25^ The existing evidence suggests differences in incident EBDs for children with different types of sleep disturbances. Therefore, a comprehensive examination of the specific types of sleep disturbances and the association with EBDs is crucial for deepening our understanding of strategies that should be prioritized to promote psychological well-being in young children.^24^

This prospective cohort study addresses these issues using data from the 2 waves of a large sample to examine how the natural history of sleep disturbances is associated with resolved and incident EBDs during preschool years. Specifically, we aimed to examine (1) whether among children with EBDs at preschool entry, resolved sleep disturbances (RSDs) are associated with resolved EBDs; (2) whether among children without EBDs at preschool entry, incident sleep disturbances (ISDs)are associated with incident EBDs; and (3) whether the association varies according to specific types of sleep disturbances.

## Methods

### Study Population

This cohort study was based on the Shanghai Children’s Health, Education and Lifestyle Evaluation–Preschool (SCHEDULE-P) study, a prospective and population-based cohort study of newly enrolled preschool children in Shanghai kindergartens.^26^ The initial sample consisted of 20 324 children from the junior class of 191 kindergartens, and their caregivers completed the survey from November 10 to 24, 2016, 2 months after school entry. The kindergartens were selected using a stratified random sampling design described in detail elsewhere.^27,28^ Of the children, 17 233 (84.8%) were followed up from April 22 to May 5, 2019, 2 months before graduation. The present study comprised a longitudinal sample with valid data on the dependent variables at both entry and graduation year, including 17 182 (84.5%) of the original 20 324 children (eFigure 2 in Supplement 1). Written informed consent was obtained from all participants at recruitment and the study was approved by the institutional review board of the Shanghai Children’s Medical Center. The study adhered to the Strengthening the Reporting of Observational Studies in Epidemiology (STROBE) reporting guideline.

### Sleep Disturbances

The Children’s Sleep Habits Questionnaire (CSHQ) is a widely used instrument for screening for sleep disturbances in children aged 4 to 10 years,^29^ and the Chinese version has been validated and extended for use in preschool children as young as 3 years.^30,31^ The instrument contains 33 items grouped into 8 subscales: Bedtime Resistance, Sleep Onset Delay, Sleep Duration, Sleep Anxiety, Night Waking, Parasomnias, Daytime Sleepiness, and Sleep-Disordered Breathing. Higher scores indicate more disturbances. Based on the original report in US children, the cutoff for the total score was set at 41.^29^ However, the cutoff of 41 might overestimate sleep disturbances in young children from some countries, especially China.^32^ We used 48 as the cutoff to screen for sleep disturbances according to a previous study,^33^ while 41 was used for the sensitivity analysis. In addition, a subscale score greater than 2 SDs above the published community control reference mean score identified specific sleep disturbance of clinical significance in each of the 8 subscale domains.^29,30^ The progression of sleep disturbances and all types of sleep disturbances were categorized into 4 groups: (1) individuals with no sleep disturbances at both time points, (2) individuals with RSDs who had sleep disturbances at the entry year but not the graduation year, (3) individuals with ISDs who had sleep disturbances at the graduation year but not the entry year, and (4) individuals with stable sleep disturbances (SSDs) who had sleep disturbances at both time points (eMethods 1 in Supplement 1).

### Emotional and Behavioral Difficulties

The Strengths and Difficulties Questionnaire was developed to screen for EBDs in children aged 3 to 17 years,^34,35,36^ including 25 items, with higher scores indicating more difficulties. Total difficulty score was dichotomized into reference (0-14) and at-risk (>14) groups, and the latter was used to define individuals with EBDs in our studies.^36,37^ A narrower definition (total score >16) of EBDs was also used for the sensitivity analysis. From entry year to graduation year, 4 exclusive groups of different transition status of EBDs were considered: individuals with no EBDs at both time points, individuals with resolved EBDs who were at risk at the entry year but not the graduation year, individuals with incident EBDs who were at risk at the graduation year but not the entry year, and individuals with stable EBDs who were at risk at both time points (eMethods 1 in Supplement 1).

### Confounding Variables

Potential covariates for the association between sleep disturbances and EBDs were identified using a directed acyclic graph of known and suspected confounders.^38^ Age, sex, screen exposure, body weight, nighttime sleep duration, marital status, maternal educational level, household income, primary caregiver, siblings, caregiving environment, parent-child interaction, and maternal emotional status were taken into account. Finally, marital status, primary caregiver, and caregiving environment were deleted to create minimally sufficient adjustment sets (eFigure 1 in Supplement 1).

### Statistical Analysis

From August 4, 2021, to October 31, 2023, data analysis was performed using Stata/SE, version 14 (StataCorp LLC). Multiple imputation with chained equations^39^ was used for the sample that had complete data on the Strengths and Difficulties Questionnaire at preschool graduation year (eMethods 2 in Supplement 1). Significance was set at a 2-sided *P* < .05. Descriptive statistics were used to characterize the sample with percentages (95% CI) and means (SD). A multilevel regression model of the group-level data (school-based) was conducted using generalized estimating equation methods^40^ to detect the association between the natural history of sleep disturbances and children’s EBD transitions after adjusting for confounders, with odds ratios (ORs) and 95% CIs being reported. The association between the transition of each type of sleep disturbance and EBD transition was also determined. After applying Bonferroni correction for multiple testing, all adjusted *P* values were compared with the significance level α = .05. A series of sensitivity analyses was conducted to examine the robustness of our findings (eTables 3-5 in Supplement 1).

## Results

### Study Sample

As summarized in Table 1, the imputed sample of 17 182 participants included 8928 boys (52.0%) and 8254 girls (48.0%), with a mean (SD) age of 3.73 (0.29) years. Compared with those excluded (n = 3142), children in the full analytic sample (n = 17 182) were more likely to have nonparental primary caregivers, no siblings, and higher maternal educational level and family income. Of the included sample, 93.1% reported all the confounders (eTable 1 in Supplement 1).

**Table 1.** Sample Characteristics of Children Included in the Study

Characteristic	Analytic sample availability^a^		OR (95% CI)	*P* value
	Included (n = 17 182)^b^	Excluded (n = 3142)^c^		
Age, mean (SD), y	3.73 (0.29)	3.72 (0.32)	NA	.06
Sex				
Boys	8928/17 182 (52.0)	1645/3142 (52.4)	0.98 (0.91-1.06)	.69
Girls	8254/17 182 (48.0)	1497/3142 (47.6)	1 [Reference]	NA
Divorced parents	506/16 856 (3.0)	123/3042 (4.0)	0.73 (0.60-0.90)	.003
Maternal educational level				
High school and below	2911/17 182 (16.9)	1358/3141 (43.2)	1 [Reference]	NA
Some college	4156/17 182 (24.2)	596/3141 (19.0)	3.25 (2.92-3.62)	<.001
Undergraduate	8136/17 182 (47.4)	906/3141 (28.8)	4.19 (3.81-4.60)	<.001
Graduate	1931/17 182 (11.2)	267/3141 (8.5)	3.37 (2.92-3.89)	<.001
Unknown	48/17 182 (0.3)	14/3141 (0.4)	1.60 (0.88-2.91)	.12
Family annual income, ¥^d^				
≤10 000	3222/17 181 (18.8)	1067/3141 (34.0)	1 [Reference]	NA
>10 000-15 000	2849/17 181 (16.6)	472/3141 (15.0)	2.00 (1.77-2.25)	<.001
>15 000-30 000	5854/17 181 (34.1)	741/3141 (23.6)	2.62 (2.36-2.90)	<.001
>30 000	4203/17 181 (24.5)	622/3141 (19.8)	2.24 (2.00-2.50)	<.001
Unknown	1053/17 181 (6.1)	239/3141 (7.6)	1.46 (1.25-1.71)	<.001
Nonparental primary caregiver	6676/17 182 (38.9)	838/3139 (26.7)	1.74 (1.60-1.90)	<.001
No siblings	12 811/17 182 (74.6)	1889/3138 (60.2)	1.94 (1.79-2.10)	<.001
Abnormal maternal emotional status^e^	2034/17 176 (11.8)	7/27 (25.9)	0.38 (0.16-0.91)	.03

Abbreviations: NA, not applicable; OR, odds ratio.

^a^ Unless otherwise indicated, data are expressed as No./total No. (%) of children. Owing to missing data, some percentages may not total 100.

^b^ Participants included in the analytic sample were those who had completed the Strengths and Difficulties Questionnaire (SDQ) at both entry to and graduation from kindergarten.

^c^ Excluded participants were children who dropped off at graduation from kindergarten and those who did not finish the SDQ.

^d^ The average conversion rate in 2016 was ¥6.64 to US $1.00.

^e^ Measured with World Health Organization Five-Item Well-Being Index at graduation year of kindergarten.

The prevalence rates of EBDs in school entry and graduation year were 27.8% and 18.7%, respectively. The prevalence of stable EBDs in children was 9.8%; no EBDs, 63.2%; resolved EBDs, 18.1%; and incident EBDs, 9.0%. The prevalence of sleep disturbances was 41.3% at entry year and 31.5% at graduation year. Among children with EBDs at the entry year, 35.0% maintained stability in the graduation year, while sleep disturbances were stable in 50.0% of children with sleep disturbances. The prevalence of each type of sleep disturbance varied. For example, the prevalence at entry year was 4.0% for night waking, 4.4% for SDB, and 11.5% for parasomnias; the prevalence at graduation year was 2.4% for night waking, 4.5% for SDB, and 6.7% for parasomnias. The prevalence was 66.5% for bedtime resistance and 53.2% for sleep anxiety at entry year; these prevalences at graduation year were 47.5% and 37.7%, respectively (eTable 2 in Supplement 1). Prevalence of SSDs in children was 19.7%; no sleep disturbances, 47.0%; RSDs, 21.5%; and ISDs, 11.8%. The prevalence of each ISD varied from 2.0% to 36.0%, while the prevalence of each RSD varied from 3.5% to 28.8% (eTable 2 in Supplement 1).

The data in Table 2 suggest that children with sleep disturbances, excessive screen time, or nonparental primary caregivers at school entry year had fewer resolved EBDs and more incident EBDs. Children with nighttime sleep duration less than 9 hours, overweight status, or divorced parents were at higher risk for incident EBDs. Children with higher maternal educational level, higher family annual income, a better caregiving environment, more frequent parent-child interaction, and fewer maternal emotional problems were more likely to have resolved EBDs and less incident EBDs.

**Table 2.** Summary Statistics of Children’s Sleep and Confounders Among Individuals With Different Transition of EBDs^a^

Characteristic	Children with EBDs at preschool entry year (n = 4785)			Children without EBDs at preschool entry year (n = 12 397)		
	Stable EBDs (n = 1676 [9.8%])	Resolved EBDs (n = 3109 [18.1%])	*P* value	No EBDs (n = 10 853 [63.2%])	Incident EBDs (n = 1544 ([9.0%])	*P* value
**Children**						
Age at enrollment, mean (SD), y^b^	3.67 (0.30)	3.72 (0.29)	<.001	3.74 (0.29)	3.74 (0.29)	.30
Sex^c^						
Boys	992 (59.2)	1683 (54.1)	<.001	5401 (49.8)	852 (55.2)	<.001
Girls	684 (40.8)	1426 (45.9)		5452 (50.2)	692 (44.8)	
Excessive screen time^c^	1436 (85.7)	2550 (82.0)	.001	7768 (71.6)	1182 (76.6)	<.001
Overweight^c^	485 (28.9)	830 (26.7)	.10	2728 (25.1)	442 (28.6)	.004
CSHQ total score >48^c^	1024 (61.1)	1643 (52.8)	<.001	3786 (34.9)	638 (41.3)	<.001
Nighttime sleep duration <9 h^c^	329 (19.6)	544 (17.5)	.07	1457 (13.4)	249 (16.1)	.004
**Family environment **						
Divorce^c^	68 (4.1)	124 (4.0)	.87	272 (2.5)	54 (3.5)	.03
Maternal educational level^c^						
Some college	502 (30.0)	819 (26.3)	.38	2455 (22.6)	380 (24.6)	.001
Undergraduate	650 (38.8)	1377 (44.3)	<.001	5404 (49.8)	705 (45.7)	<.001
Graduate	72 (4.3)	230 (7.4)	<.001	1481 (13.6)	148 (9.6)	<.001
Unknown	9 (0.5)	11 (0.4)	.63	21 (0.2)	7 (0.5)	.26
Family annual income,¥^c^^,^^d^						
>10 000-15 000	357 (21.3)	551 (17.7)	.80	1653 (15.2)	288 (18.7)	.26
>15 000-30 000	501 (29.9)	1039 (33.4)	<.001	3805 (35.1)	509 (33.0)	<.001
>30 000	250 (14.9)	625 (20.1)	<.001	3008 (27.7)	320 (20.7)	<.001
Unknown	96 (5.7)	181 (5.8)	.11	678 (6.2)	98 (6.3)	.02
Nonparental primary caregiver^c^	751 (44.8)	1274 (41.0)	.01	4014 (37.0)	637 (41.3)	.001
No siblings^c^	1283 (76.6)	2361 (75.9)	.64	8001 (73.7)	1166 (75.5)	.13
ICCE score^c^^,^^e^						
10	291 (17.4)	463 (14.9)	.27	1074 (9.9)	215 (13.9)	.39
11	377 (22.5)	688 (22.1)	.007	2066 (19.0)	296 (19.2)	<.001
12	362 (21.6)	800 (25.7)	<.001	3332 (30.7)	447 (29.0)	<.001
13	239 (14.3)	581 (18.7)	<.001	3387 (31.2)	367 (23.8)	<.001
CPCIS score^c^^,^^f^						
1.01-2.00	534 (31.9)	869 (28.0)	.29	1805 (16.6)	348 (22.5)	.002
2.01-3.00	630 (37.6)	1140 (36.7)	.06	3625 (33.4)	560 (36.3)	<.001
3.01-4.00	338 (20.2)	767 (24.7)	.001	3828 (35.3)	421 (27.3)	<.001
4.01-5.00	58 (3.5)	169 (5.4)	<.001	1340 (12.3)	138 (8.9)	<.001
Abnormal maternal emotional status^c^	374 (22.3)	382 (12.3)	<.001	985 (9.1)	294 (19.0)	<.001

Abbreviations: CPCIS, Chinese Parent-Child Interaction Scale; CSHQ, Children’s Sleep Habits Questionnaire; EBD, emotional and behavioral difficulty; ICCE, Index of Child Care Environment.

^a^ Unless otherwise indicated, data are expressed as No. (%) of children.

^b^ Evaluated with independent samples 2-tailed *t* test.

^c^ Evaluated with χ^2^ tests.

^d^ The average conversion rate in 2016 was ¥6.64 to US $1.00.

^e^ Scores range from 0 to 13, with higher scores indicating better quality of caring environment. Comparisons are scores 9 or less.

^f^ Scores range from 0 to 5.00, with higher scores indicating more parent-child interaction. Comparisons are scores 1.00 or less.

### Association of Natural History of Sleep Disturbances With Resolved and Incident EBDs

Data in Table 3 suggest that among children with EBDs at entry year, both ISDs and SSDs were associated with a decreased odds of resolved EBDs. After adjustment for confounders, the OR of resolved EBDs was lower among the groups with ISDs (OR, 0.50 [95% CI, 0.41-0.62]; *P* < .001) and SSDs (OR, 0.47 [95% CI, 0.40-0.56]; *P* < .001) compared with the group with no sleep disturbances. Children with RSDs were also less likely to have resolved EBDs (OR, 0.78 [95% CI, 0.66-0.92]; *P* = .003) compared with the group with no sleep disturbances. In contrast, the groups with no sleep disturbances (OR, 2.11 [95% CI, 1.80-2.47]; *P* < .001) and RSDs (OR, 1.64 [95% CI, 1.39-1.93]; *P* < .001) were more likely to have resolved EBDs compared with the SSD group (eFigure 3 in Supplement 1). In Table 3, after adjusting for confounders, incident EBDs were more likely to occur in the ISD group (OR, 2.58 [95% CI, 2.22-3.01]; *P* < .001) and the SSD group (OR, 2.29 [95% CI, 1.98-2.64]; *P* < .001) than in the group with no sleep disturbances, but not in the group with RSDs.

**Table 3.** Association Between Sleep Status Transition From Preschool Entry Year to Graduation Year and Transition of EBDs

Sleep status	Unadjusted model		Adjusted model^a^	
	OR (95% CI)	*P* value	OR (95% CI)	*P* value
**Resolved EBDs in children with EBDs at preschool entry year**				
No sleep disturbances	1 [Reference]	NA	1 [Reference]	NA
Incident sleep disturbances	0.48 (0.39-0.59)	<.001	0.50 (0.41-0.62)	<.001
Resolved sleep disturbances	0.78 (0.66-0.91)	.002	0.78 (0.66-0.92)	.003
Stable sleep disturbances	0.46 (0.39-0.53)	<.001	0.47 (0.40-0.56)	<.001
**Incident EBDs in children without EBDs at preschool entry year**				
No sleep disturbances	1 [Reference]	NA	1 [Reference]	NA
Incident sleep disturbances	2.79 (2.40-3.24)	<.001	2.58 (2.22-3.01)	<.001
Resolved sleep disturbances	1.16 (1.00-1.36)	.06	1.10 (0.94-1.29)	.31
Stable sleep disturbances	2.44 (2.12-2.81)	<.001	2.29 (1.98-2.64)	<.001

Abbreviation: EBD, emotional and behavioral difficulty; NA, not applicable; OR, odds ratio.

^a^ Adjusted for sex, age, maternal educational level, family annual income, siblings, Chinese Parent-Child Interaction Scale total scores, nighttime sleep duration, screen exposure time, weight status measured at preschool entry year, and maternal emotional status measured at preschool graduation year. Sleep disturbances are defined as Children’s Sleep Habits Questionnaire total scores of greater than 48.

### Association of Natural Transition of Different Types of Sleep Disturbances With Resolved and Incident EBDs

**Figure 1.**
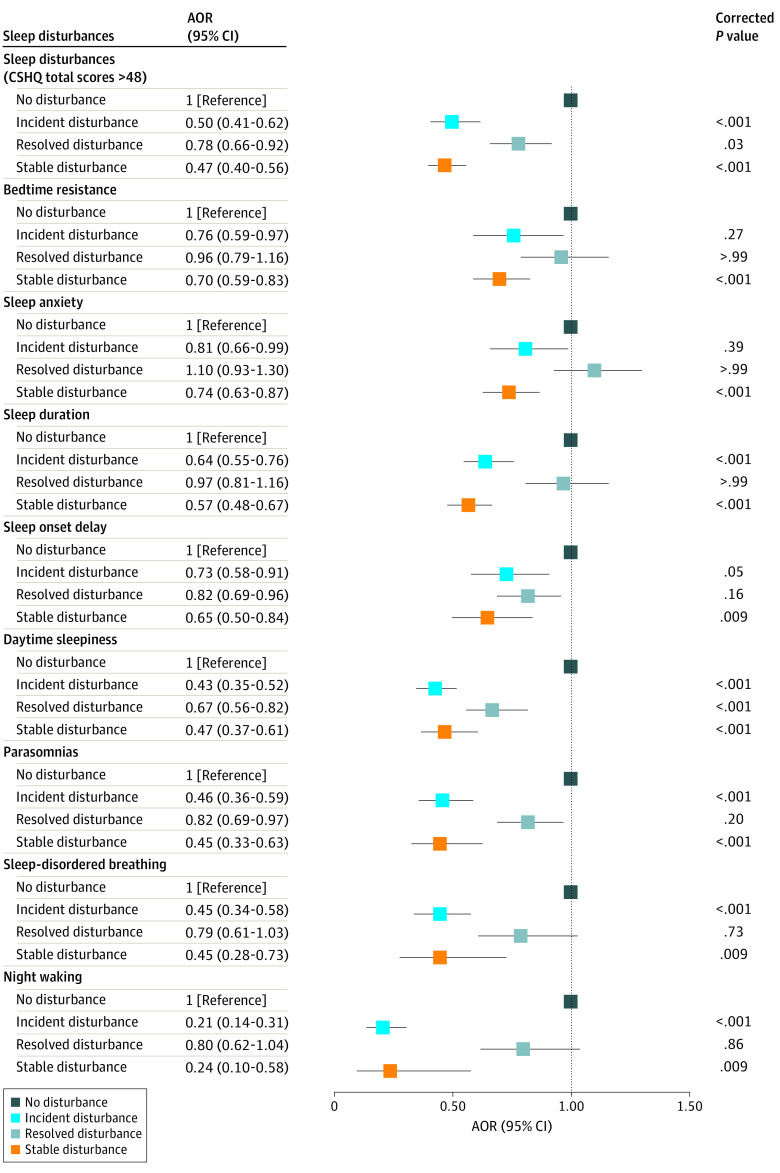
Odds of Resolved Emotional and Behavioral Difficulties for Children With Transition of Different Sleep Disturbances Comparisons are with children without sleep disturbances. All the *P* values are presented after Bonferroni multiple testing adjustments. AOR indicates adjusted odds ratio; CSHQ, Children’s Sleep Habits Questionnaire.

Figure 1 shows the association between the natural transition of each type of sleep disturbance and resolved EBDs. Compared with children with no night-waking disturbance, those with SSDs (OR, 0.24 [95% CI, 0.10-0.58]; *P* = .009) or ISDs (OR, 0.21 [95% CI, 0.14-0.31]; *P* < .001) were less likely to have resolved EBDs. The likelihood of resolved EBDs increased for children with resolved bedtime resistance (OR, 1.37 [95% CI, 1.18-1.58]; *P* < .001), sleep anxiety (OR, 1.48 [95% CI, 1.27-1.74]; *P* < .001), sleep duration (OR, 1.71 [95% CI, 1.40-2.09]; *P* < .001), and parasomnias (OR, 1.80 [95% CI, 1.26-2.58]; *P* = .009) compared with those with SSDs (eFigure 3 in Supplement 1).

**Figure 2.**
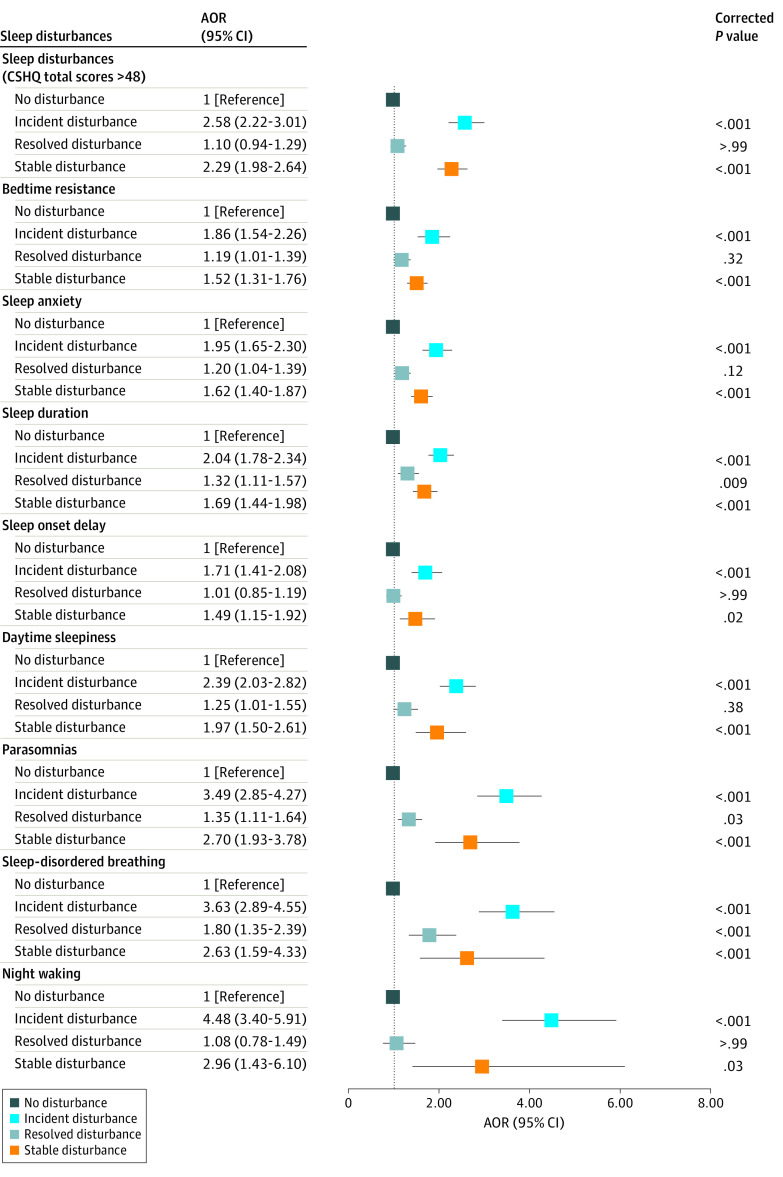
Odds of Incident Emotional and Behavioral Difficulties for Children With Transition of Different Sleep Disturbances Comparisons are with children without sleep disturbances. All the *P* values are presented after Bonferroni multiple testing adjustments. AOR indicates adjusted odds ratio; CSHQ, Children’s Sleep Habits Questionnaire.

Figure 2 shows the association between the natural transition of each type of sleep disturbance and incidence of EBDs. Compared with the group with no sleep disturbances, SSDs and ISDs of any type were associated with an increased likelihood of incident EBDs. For example, the risk for incident EBDs increased for children with incident parasomnias (OR, 3.49 [95% CI, 2.85-4.27]; *P* < .001), SDB (OR, 3.63 [95% CI, 2.89-4.55]; *P* < .001), and night waking disturbances (OR, 4.48 [95% CI, 3.40-5.91]; *P* < .001).

### Sensitivity Analyses

Using the cutoff value of 41 for sleep disturbances (eTable 3 in Supplement 1), the group with RSDs (17.4%) was associated with a higher risk of incident EBDs compared with the group with no sleep disturbances (OR, 1.50 [95% CI, 1.12-2.00]; *P* = .007) but not with resolved EBDs. Using a narrow definition of EBDs, the results of the association between sleep transition and transition of EBDs were essentially the same (eTable 4 in Supplement 1). Furthermore, generalized estimating equation modeling was performed in data sets in which mothers had stable emotional status and the results remained unchanged (eTable 5 in Supplement 1).

## Discussion

The current cohort study is the first, to our knowledge, to examine how the natural history of sleep disturbances is associated with resolved and incident EBDs in a large sample of preschool-aged children. We found that ISDs and SSDs were associated with decreased resolved EBDs and increased incident EBDs, whereas RSDs were associated with more resolved EBDs as well as never having sleep disturbances. Sensitivity analyses ensured the robustness of our findings.

Incident and stable sleep disturbances were associated with an increased risk of incident EBDs, but decreased resolved EBDs. A high incidence (12.5%) of EBDs among preschool graduates who exhibited no EBDs at preschool entry warrants attention. Once EBDs appear at the preschool graduation year, children may be not psychologically and socially ready for the next educational step. Children with RSDs showed a higher risk of incident EBDs when using 41 as the cutoff value on the CSHQ. However, RSDs (17.4% at the cutoff value of 41) may have been underestimated due to the potential overestimation of sleep disturbances.^32^ Children with RSDs defined using a cutoff value of 41 may still experience troubled sleep and may be more likely to have incident EBDs. The link of disturbed sleep with psychosocial dysregulation has been suggested,^21^ based on several possible underlying mechanisms, including dysregulation of the hypothalamic-pituitary-adrenal axis.^41^ These mechanisms decrease daytime vigilance, which results in agitation and affective disturbances.^42^ Therefore, prevention and intervention of ISDs and SSDs are indisputably important for school readiness and mental health promotion.

Our study found that for those children who had EBDs at baseline, RSD was associated with resolved EBDs. A randomized clinical trial performed among school-aged children^43^ successfully improved both sleep disturbances and EBDs through targeted interventions based on sleep disturbance screening. However, a separate translational study failed to replicate the benefits of the initial trial.^44^ The age of the targeted population, different strategies, and the dose of intervention may determine the benefits. Sleep intervention trials^44^ including preschool-aged children found the benefit for psychosocial well-being was limited. Our results indicate that compared with the SSD group, the targeted population who had sleep disturbances at baseline was more likely to have resolved EBDs (eFigure 3 in Supplement 1) with RSDs. Furthermore, for those without EBDs at baseline, the advantage diminished. In summary, successful parental sleep management, similar to natural RSDs observed in our study, may help improve both sleep disturbances and EBDs.

The varying risk of different types of sleep disturbances linked to resolved and incident EBDs warrants attention. In our study, the most robust findings were for night waking, SDB, and parasomnias. Interestingly, these disturbances were less common in preschool children, with prevalence rates of 4.0% for night waking, 4.4% for SDB, and 11.5% for parasomnias at the entry year and 2.4% for night waking, 4.5% for SDB, and 6.7% for parasomnias at the graduation year. Night waking is usually a normative part of early development, except when accompanied by prolonged awakening requiring parental assistance.^45^ Prolonged and recurrent signaled night waking can persist in children aged 4 to 6 years.^46^ In line with previous studies,^47,48^ young children with incident night waking in our study had 300% increased odds of having incident EBDs. Additionally, resolved bedtime resistance, sleep anxiety, irregular sleep duration, and parasomnias were found to be associated with the resolved EBDs. Therefore, our study asserted that precise interventions for sleep disturbances, which call for the specified screening and tailored treatment of the referred sleep disturbances among preschool-aged children, might be promising to improve their psychosocial well-being.

### Limitations

Several limitations should be considered when interpreting the findings of this study. First, due to the large-scale and population-based nature of the study, clinical interviews were not used to assess sleep disturbances and EBDs. The sleep disturbances and EBDs defined in our study may not align with diagnosed problems. It is possible that the biases associated with parental report may have masked associations. However, a parent-reported problem was always the antecedent signal for diagnosing and seeking treatment for these concerns, which correlated well with objective measures of sleep disturbances.^49^ Furthermore, the results remained consistent even after excluding those mothers with abnormal emotional status, who may overestimate children’s sleep disturbances and EBDs. Second, our study attempted to answer whether different sleep disturbances play a different role in resolved EBDs and incident EBDs using the 8 subscales of the CSHQ. Although the CSHQ targets symptoms of common pediatric sleep disorders and is based on the International Classification of Sleep Disorders, circadian rhythm problems and sleep-related movement were missed, which calls for future studies, especially among preschool-aged children. Third, other potential confounding factors, such as children’s total sleep duration, temperament, genetic susceptibility, caregiver’s sleep problems, and time-varying factors were not considered. Fourth, the epidemiological design used herein cannot determine causality. Bidirectional association should also be considered.^50^ Reverse analysis showed similar but weaker associations. Fifth, the SCHEDULE-P study is representative of the population of Shanghai, one of China’s most developed cities. As in most cohort studies, attrition may have occurred over time, leading to potential selection bias, despite multiple imputation. The high socioeconomic status and selection bias of the included data set may limit generalization to other populations, especially in low socioeconomic groups.

## Conclusions

To our knowledge, this cohort study is the first to find an association between the natural history of sleep disturbances and resolved and incident EBDs, which opens the way for targeted intervention for various sleep disturbances to improve the well-being of preschool-aged children. Nevertheless, according to our findings, not only behavioral sleep disturbances but also sleep disturbances with low incidence require great attention to screening and comprehensive intervention for the sake of cost-effective improvements in resolving children’s EBDs.

Our findings support the consistent integration of questions about sleep into routine developmental screenings in school and primary care contexts. Furthermore, to determine whether addressing these comorbidities can prevent their progression and effects on child well-being, interventions that target symptoms of both sleep disturbance and EBDs are needed.

## Notes

Supplement 1. **eMethods 1.** Choice of Primary Measures **eFigure 1.** Directed Acyclic Graph **eMethods 2.** Statistical Analysis **eFigure 2.** Flow Diagram of Included Participants **eTable 1.** Summary Statistics of Main Variables in the Observed Complete-Case Data Set (n = 16 000) and the Imputed Data Set (n = 17 182) **eTable 2.** Prevalence of Each Sleep Disturbance **eTable 3.** Association Between Sleep Status Transition From Preschool Year of Entry to Year of Graduation and Transition of EBDs **eTable 4.** Association Between Sleep Status Transition From Preschool Year of Entry to Year of Graduation and Transition of EBDs (Narrow Definition) **eTable 5.** Association Between Sleep Status Transition From Preschool Year of Entry to Year of Graduation and Transition of EBDs in Data Sets in Which Mothers Had Normal Emotional Status **eFigure 3.** The Odds Ratio of Resolved Emotional and Behavioral Difficulties (EBDs) for Children With the Transition of Various Sleep Disturbances Compared With Children With Stable Sleep Disturbances
eReferences

Supplement 2.
Data Sharing Statement
