# Supplementary material for: Sleep Disturbances and Emotional and Behavioral Difficulties Among Preschool-Aged Children
Source: JAMA Netw Open. 2023 Dec 14;6(12):e2347623. doi: 10.1001/jamanetworkopen.2023.47623 (PMC10722331; doi:10.1001/jamanetworkopen.2023.47623)
Supplement: Supplement 1. — eMethods 1. Choice of Primary Measures eFigure 1. Directed Acyclic Graph eMethods 2. Statistical Analysis eFigure 2. Flow Diagram of Included Participants eTable 1. Summary Statistics of Main Variables in the Observed Complete-Case Data Set (n = 16 000) and the Imputed Data Set (n = 17 182) eTable 2. Prevalence of Each Sleep Disturbance eTable 3. Association Between Sleep Status Transition From Preschool Year of Entry to Year of Graduation and Transition of EBDs eTable 4. Association Between Sleep Status Transition From Preschool Year of Entry to Year of Graduation and Transition of EBDs (Narrow Definition) eTable 5. Association Between Sleep Status Transition From Preschool Year of Entry to Year of Graduation and Transition of EBDs in Data Sets in Which Mothers Had Normal Emotional Status eFigure 3. The Odds Ratio of Resolved Emotional and Behavioral Difficulties (EBDs) for Children With the Transition of Various Sleep Disturbances Compared With Children With Stable Sleep Disturbances eReferences [file jamanetwopen-e2347623-s001.pdf]

Supplementary Online Content

Deng Y, Zhang Z, Gui Y, et al. Sleep disturbances and emotional and behavioral difficulties among preschool-aged children. *JAMA Netw Open*. 2023;6(12):e2347623. doi:10.1001/jamanetworkopen.2023.47623

eMethods 1. Choice of Primary Measures
eFigure 1. Directed Acyclic Graph
eMethods 2. Statistical Analysis
eFigure 2. Flow Diagram of Included Participants
eTable 1. Summary Statistics of Main Variables in the Observed Complete-Case Data Set (n = 16 000) and the Imputed Data Set (n = 17 182)
eTable 2. Prevalence of Each Sleep Disturbance
eTable 3. Association Between Sleep Status Transition From Preschool Year of Entry to Year of Graduation and Transition of EBDs
eTable 4. Association Between Sleep Status Transition From Preschool Year of Entry to Year of Graduation and Transition of EBDs (Narrow Definition)
eTable 5. Association Between Sleep Status Transition From Preschool Year of Entry to Year of Graduation and Transition of EBDs in Data Sets in Which Mothers Had Normal Emotional Status
eFigure 3. The Odds Ratio of Resolved Emotional and Behavioral Difficulties (EBDs) for Children With the Transition of Various Sleep Disturbances Compared With Children With Stable Sleep Disturbances
eReferences

This supplementary material has been provided by the authors to give readers additional information about their work.

© 2023 Deng Y et al. *JAMA Network Open*.

eMethods. Choice of Primary Measures

Sleep Disturbances

The reliability and validity of the Mandarin version of the Children's Sleep Habits Questionnaire (CSHQ) have been demonstrated in several studies.[1,2] Parents rate the frequency for each item on a 3-point Likert Scale based on observations of their children during the past recent week or a typical week: "usually" (5-7 times per week), "sometimes" (2-4 times per week), and "rarely" (0-1 time per week). A higher total score on the CSHQ indicates greater sleep disturbances. In this study, the CSHQ yielded an acceptable Cronbach's alpha coefficient of 0.74 at the year of entry, 0.77 at the year of graduation. Furthermore, the progression of specific sleep disturbances (8 types of sleep disturbances) was categorized into 4 groups: individuals with no sleep disturbances at both time points, individuals with resolved sleep disturbances (RSDs) who had sleep disturbances in the year of entry but not in the year of graduation, individuals with incident sleep disturbances (ISDs) who had sleep disturbances in the year of graduation but not in the year of entry, individuals with stable sleep disturbances (SSDs) who had sleep disturbances at both time points.

Emotional and Behavioral Disturbances (EBDs)

Using the Strengths and Difficulties Questionnaire, parents rate the children's behavior in the past six months on a 3-point Likert scale: not true=0, somewhat true=1, and definitely true=2. The total difficulties score is calculated by summing the scores of the 4 difficulties subscale scores. In this study, the Cronbach's alpha coefficient for the full scale was 0.80 at year of entry and 0.83 at graduation year.

Confounding factors

We examined several variables as potential confounders, such as child characteristics, socioemotional status (including maternal educational level, and family annual income), maternal psychological status and caring environment. Initially, we calculated the duration of a child's nightly sleep (NSD) on workdays (NSD-workday) and on free days (NSD-freeday) as the number of hours between bedtime and wake time. NSD. Nighttime sleep duration was categorized into two group (less than 9 hours and 9 hours or more).[3] Children's weight status was assessed using the z-scores of their Body Mass Index (BMI).[4] Daily screen time exceeding 1 hours per day was considered as excessive.[5] Demographic information such as the parents' marital status (divorced or not), the child's gender, age at school entry, primary caregiver (parent or other), and number of siblings was collected. The Index of Child Caring Environment (ICCE) was utilized to assess the quality of caring environment and categorized into 5 rankings (raw scores ≤1, 1.01-2, 2.01-3, 3.01-4, 4.01-5), with higher rankings indicated better quality.[6] Moreover, we evaluated parent-child interactions using the China Parent-Child Interaction Scores (CPCIS),[7] a questionnaire that gauges the quantity of the most prevalent activities related to learning, reading, recreation, and environment interaction. Total scores were divided into 5 ranks (raw scores ≤9, =10, =11, =12, =13) based on the frequency of parent–child interactive activities, with higher scores indicating more interaction. All these confounders were evaluated at the year of entry except for maternal psychological status, which was examined using the World Health Organization's five-item well-being index (WHO-5) and separated into abnormal (total scores<13) and normal (total scores ≤13) groups at the year of graduation.[8]

© 2023 Deng Y et al. *JAMA Network Open*.

eFigure 1. Directed Acyclic Graph

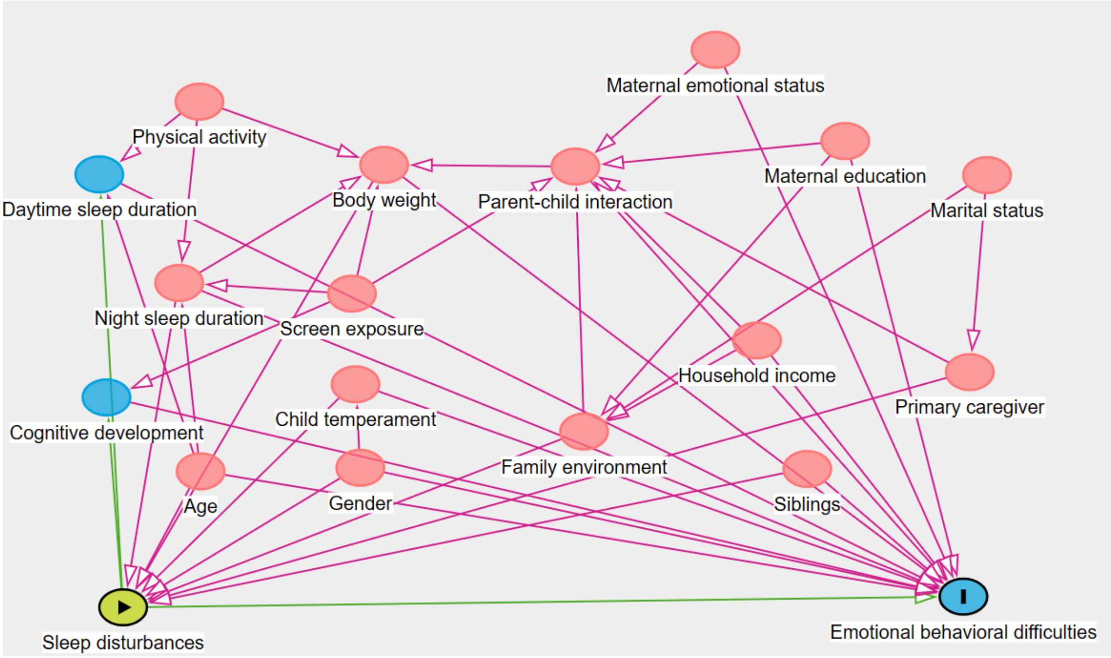

eMethods 2. Statistical Analysis

Multiple imputation

To address potential bias due to attrition and to handle missing values, multiple imputation with chained equations[9] was used for the sample that had complete data on the SDQ at graduation year of preschool (17 182). For the current analyses, 50 imputed datasets were created using information from the included variables with information from 4 additional variables associated with observed data and missingness, i.e., birth weight status, breastfeeding, kindergartens, and districts.

Sensitive analysis

First, the cut-off of 41 for the CSHQ total score was used to act as a broad definition of sleep disturbances (eTable 3 in Supplement 3). To examine the association between sleep disturbances and more severe EBDs, we used a narrower definition of abnormal in EBDs (eTable 4 in Supplement 3). In addition, we conducted the regression in the datasets in which mothers have a normal emotional status, taking into account the possible reporting bias for children's sleep disturbances and EBDs (eTable 5 in Supplement 3).

© 2023 Deng Y et al. *JAMA Network Open*.

eFigure 2. Flow Diagram of Included Participants

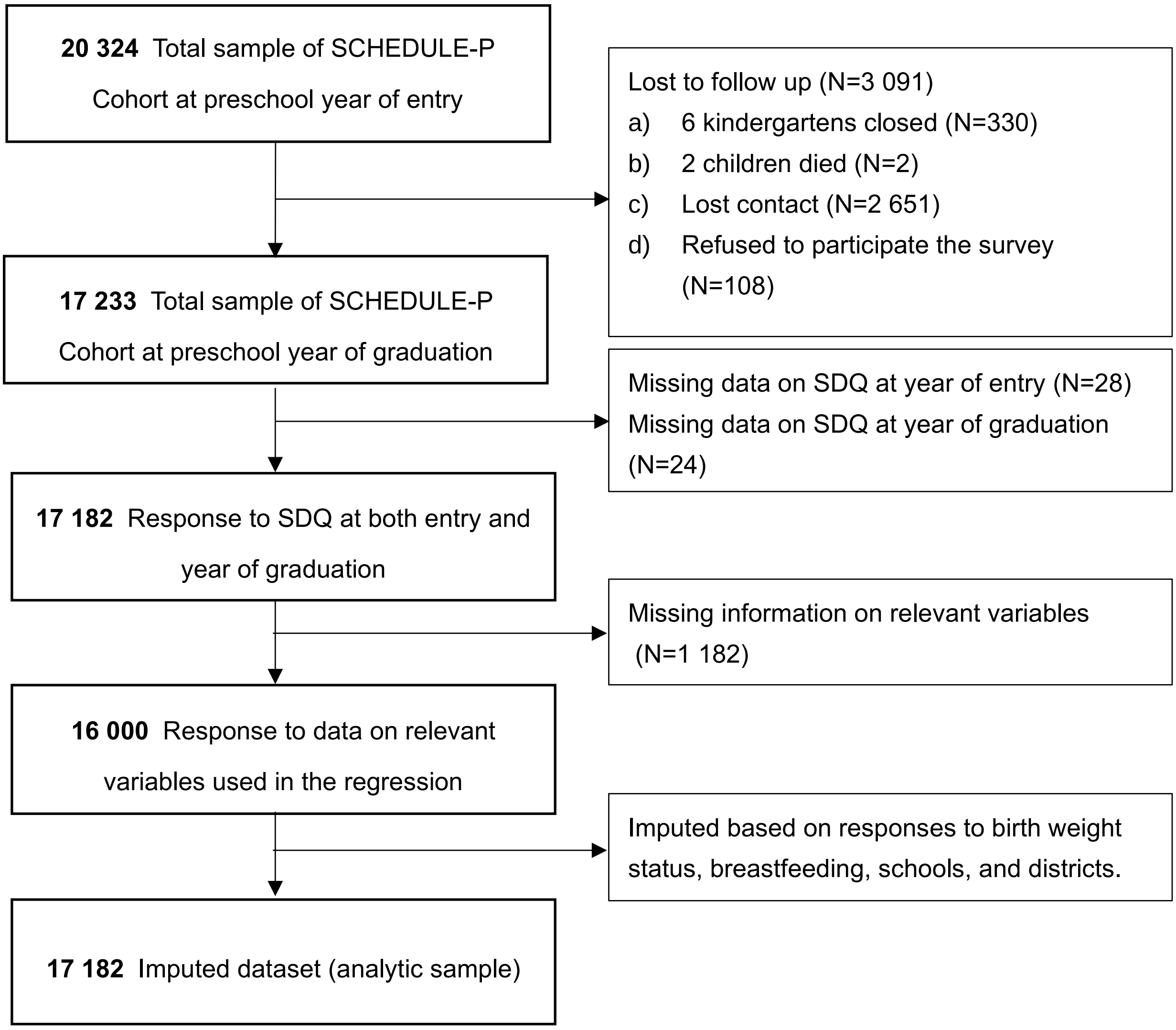

© 2023 Deng Y et al. *JAMA Network Open*.

eTable 1. Summary Statistics of Main Variables in the Observed Complete-Case Data Set (n = 16 000) and the Imputed Data Set (n = 17 182)

	Missing data % (n)	Observed data (%) /mean (SD)	Imputed data (%) /mean (SD)
Variables			
Divorce	1.9 (326)	3	3
Maternal educational level			
High school and below	0 (0)	16.9	16.9
Some college		24.2	24.2
Undergraduate		47.4	47.4
Graduate		11.2	11.2
Unknown		0.3	0.3
Family annual income, ¥ [a]			
≤10 000	0.01 (1)	18.8	18.8
>10 000 to 15 000		16.6	16.6
>15 000 to 30 000		34.1	34.1
>30 000		24.5	24.5
Unknown		6.1	6.1
Non parental primary caregiver	0 (0)	38.9	38.9
Single child	0 (0)	75.4	75.3
Index of Child Care Environment			
≤9	1.57 (269)	12.6	12.8
10		11.9	11.9
11		20.0	19.9
12		28.8	28.8
13		26.7	26.6
Chinese Parent-Child Interaction Scores			
≤1	0.18 (31)	3.5	3.6
1.01-2		20.7	20.7
2.01-3		34.7	34.7
3.01-4		31.2	31.1
4.01-5		9.9	9.9
Screen exposure for more than1 hour	0.39 (67)	75.3	75.3
Overweight	1.74 (299)	26.0	26.1
Abnormal maternal emotional status	0.03 (6)	11.8	11.8
Nighttime sleep duration	0.63 (108)	9.51 (0.58)	9.52 (0.63)
CSHQ total scores			
Sleep at preschool year of entry	0.03 (6)	47.74 (6.38)	48.35 (6.92)
Sleep at preschool year of graduation	0	46.01 (6.71)	46.83 (7.39)

[a]The average conversion rate in 2016 was 6.64 yuan to $1.00 US.

Abbreviation: CSHQ, Children's Sleep Habits Questionnaire.

© 2023 Deng Y et al. *JAMA Network Open*.

eResults

eTable 2. Prevalence of Each Types of Sleep Disturbance

	N. (%)					
	Sleep disturbances at school entrance Year	Sleep disturbances at graduate Year	No sleep disturbances	Incident sleep disturbances	Resolved sleep disturbances	Stable sleep disturbances
Sleep disturbances (>48)	7096 (41.3)	5412 (31.5)	8076 (47.0)	2027 (11.8)	3694 (21.5)	3385 (19.7)
Sleep disturbances (>41)	14484 (84.4)	12732 (74.1)	1460 (8.5)	1237 (7.2)	2990 (17.4)	11495 (66.9)
Subscales of sleep disturbances						
Bedtime resistance	11426 (66.5)	8161 (47.5)	4072 (23.7)	1684 (9.8)	4948 (28.8)	6478 (37.7)
Sleep anxiety	9141 (53.2)	6478 (37.7)	6014 (35.0)	2027 (11.8)	4691 (27.3)	4450 (25.9)
Sleep duration	5000 (29.1)	5808 (33.8)	8986 (52.3)	3196 (18.6)	2388 (13.9)	2612 (15.2)
Sleep onset delay	3076 (17.9)	1873 (10.9)	12972 (75.5)	1134 (6.6)	2337 (13.6)	739 (4.3)
Daytime sleepiness	1924 (11.2)	2096 (12.2)	13763 (80.1)	1495 (8.7)	1323 (7.7)	601 (3.5)
Parasomnias	1976 (11.5)	1151 (6.7)	14399 (83.8)	808 (4.7)	1632 (9.5)	344 (2.0)
Sleep disordered breathing	756 (4.4)	773 (4.5)	15807 (92.0)	6186 (36.0)	601 (3.5)	155 (0.9)
Night waking	687 (4.0)	412 (2.4)	16151 (94.0)	344 (2.0)	619 (3.6)	69 (0.4)

© 2023 Deng Y et al. *JAMA Network Open*.

eTable 3. Association Between Sleep Status Transition From Preschool Year of Entry to Year of Graduation and Transition of EBDs

	**Baseline Model**		**Adjusted Model[a]**	
**Variable**	**OR (95%CI)**	***P* value**	**OR (95%CI)**	***P* value**
**Resolved EBDs in children with EBDs at preschool year of entry**				
No sleep disturbances	ref	NA	ref	NA
Incident sleep disturbances	0.49 (0.31 , 0.77)	.002	0.47 (0.30 , 0.76)	.002
Resolved sleep disturbances	0.90 (0.60 , 1.36)	.63	0.89 (0.59 , 1.34)	.57
Stable sleep disturbances	0.36 (0.25 , 0.52)	<.001	0.37 (0.25 , 0.54)	<.001
**Incident EBDs in children without EBDs at preschool year of entry**				
No sleep disturbances	ref	NA	ref	NA
Incident sleep disturbances	3.05 (2.25 , 4.13)	<.001	2.83 (2.09 , 3.85)	<.001
Resolved sleep disturbances	1.59 (1.19 , 2.12)	.002	1.50 (1.12 , 2.00)	.007
Stable sleep disturbances	3.14 (2.43 , 4.05)	<.001	2.79 (2.15 , 3.62)	<.001

Abbreviation: EBDs, emotional and behavioral difficulties; NA, not applicable; OR, odds ratio.

[a]adjusted for sex, age, maternal educational level, family annual income, siblings, Chinese Parent-Child Interaction Scale total scores, nighttime sleep duration, screen exposure time, weight status measured at preschool year of entry, and maternal emotional status measured at preschool year of graduation.

Sleep disturbances, Children's Sleep Habits Questionnaire total scores >41.

© 2023 Deng Y et al. *JAMA Network Open*.

eTable 4. Association Between Sleep Status Transition From Preschool Year of Entry to Year of Graduation and Transition of EBDs (Narrow Definition)

	Baseline Model		Adjusted Model[a]	
**Variable**	**OR (95%CI)**	***P* value**	**OR (95%CI)**	***P* value**
**Resolved EBDs in children with EBDs at preschool year of entry**				
No sleep disturbances	ref	NA	ref	NA
Incident sleep disturbances	0.47 (0.35 , 0.63)	<.001	0.50 (0.37 , 0.67)	<.001
Resolved sleep disturbances	0.77 (0.61 , 0.98)	.03	0.79 (0.62 , 1.01)	.06
Stable sleep disturbances	0.39 (0.32 , 0.49)	<.001	0.42 (0.34 , 0.53)	<.001
**Incident EBDs in children without EBDs at preschool year of entry**				
No sleep disturbances	ref	NA	ref	NA
Incident sleep disturbances	2.71 (2.31 , 3.18)	<.001	2.55 (2.17 , 3.00)	<.001
Resolved sleep disturbances	1.13 (0.96 , 1.34)	.14	1.08 (0.91 , 1.28)	.36
Stable sleep disturbances	2.41 (2.08 , 2.79)	<.001	2.26 (1.91 , 2.63)	<.001

Abbreviation: EBDs, emotional and behavioral difficulties; NA, not applicable; OR, odds ratio.

[a]adjusted for sex, age, maternal educational level, family annual income, siblings, Chinese Parent-Child Interaction Scale total scores, nighttime sleep duration, screen exposure time, weight status measured at preschool year of entry, and maternal emotional status measured at preschool year of graduation.

Sleep disturbances, Children's Sleep Habits Questionnaire total scores >48;

© 2023 Deng Y et al. *JAMA Network Open*.

eTable 5. Association Between Sleep Status Transition From Preschool Year of Entry to Year of Graduation and Transition of EBDs in Data Sets in Which Mothers Had Normal Emotional Status

	Baseline Model		Adjusted Model[a]	
**Variable**	**OR (95%CI)**	***P* value**	**OR (95%CI)**	***P* value**
**Resolved EBDs in children with EBDs at preschool year of entry**				
No sleep disturbances	ref	NA	ref	NA
Incident sleep disturbances	0.48 (0.38 , 0.60)	<.001	0.49 (0.39 , 0.61)	<.001
Resolved sleep disturbances	0.82 (0.68 , 0.97)	.03	0.81 (0.67 , 0.97)	.02
Stable sleep disturbances	0.50 (0.43 , 0.60)	<.001	0.48 (0.41 , 0.58)	<.001
**Incident EBDs in children without EBDs at preschool year of entry**				
No sleep disturbances	ref	NA	ref	NA
Incident sleep disturbances	2.78 (2.35 , 3.28)	<.001	2.68 (2.27 , 3.17)	<.001
Resolved sleep disturbances	1.13 (0.96 , 1.34)	.15	1.10 (0.94 , 1.32)	.25
Stable sleep disturbances	2.43 (2.08 , 2.83)	<.001	2.42 (2.06 , 2.85)	<.001

Abbreviation: EBDs, emotional and behavioral difficulties; NA, not applicable; OR, odds ratio.

[a]adjusted for sex, age, maternal educational level, family annual income, siblings, Chinese Parent-Child Interaction Scale total scores, nighttime sleep duration, screen exposure time, weight status measured at preschool year of entry, and maternal emotional status measured at preschool year of graduation.

Sleep disturbances, Children's Sleep Habits Questionnaire total scores >48;

© 2023 Deng Y et al. *JAMA Network Open*.

eFigure 3. Odds of Resolved Emotional and Behavioral Difficulties for Children With Transition of Different Sleep Disturbances

Sleep disturbances	Adjusted OR (95% CI)	Corrected P value
**Sleep disturbances (CSHQ total scores>48)**		
Stable disturbance	1 [Reference]	
No disturbance	1.11 (1.80 , 2.47)	<0.001
Incident disturbance	1.06 (0.87 , 1.31)	0.95
Resolved disturbance	1.64 (1.39 , 1.93)	<0.001
**Sleep disturbances (CSHQ total scores>41)**		
Stable disturbance	1 [Reference]	
No disturbance	2.71 (1.86 , 3.93)	<0.001
Incident disturbance	1.28 (0.95 , 1.73)	>.99
Resolved disturbance	2.40 (1.96 , 2.94)	<0.001
**Bedtime resistance**		
Stable disturbance	1 [Reference]	
No disturbance	1.43 (1.20 , 1.70)	<0.001
Incident disturbance	1.09 (0.87 , 1.35)	>.99
Resolved disturbance	1.37 (1.18 , 1.58)	<0.001
**Sleep anxiety**		
Stable disturbance	1 [Reference]	
No disturbance	1.35 (1.15 , 1.58)	<0.001
Incident disturbance	1.09 (0.89 , 1.33)	>.99
Resolved disturbance	1.48 (1.27 , 1.74)	<0.001
**Sleep duration**		
Stable disturbance	1 [Reference]	
No disturbance	1.77 (1.50 , 2.08)	<0.001
Incident disturbance	1.14 (0.94 , 1.37)	>.99
Resolved disturbance	1.71 (1.40 , 2.09)	<0.001
**Sleep onset delay**		
Stable disturbance	1 [Reference]	
No disturbance	1.54 (1.19 , 1.99)	0.009
Incident disturbance	1.12 (0.81 , 1.55)	>.99
Resolved disturbance	1.26 (0.94 , 1.68)	>.99
**Daytime sleepiness**		
Stable disturbance	1 [Reference]	
No disturbance	2.11 (1.63 , 2.72)	<0.001
Incident disturbance	0.91 (0.67 , 1.23)	>.99
Resolved disturbance	1.42 (1.05 , 1.92)	0.21
**Parasomnias**		
Stable disturbance	1 [Reference]	
No disturbance	2.20 (1.58 , 3.06)	<0.001
Incident disturbance	1.02 (0.68 , 1.51)	>.99
Resolved disturbance	1.80 (1.26 , 2.58)	0.009
**Sleep disordered breathing**		
Stable disturbance	1 [Reference]	
No disturbance	2.20 (1.38 , 3.52)	0.009
Incident disturbance	0.98 (0.58 , 1.67)	>.99
Resolved disturbance	1.75 (1.03 , 2.96)	0.33
**Night waking**		
Stable disturbance	1 [Reference]	
No disturbance	4.13 (1.72 , 9.90)	0.009
Incident disturbance	0.86 (0.33 , 2.25)	>.99
Resolved disturbance	3.31 (1.34 , 8.20)	0.09

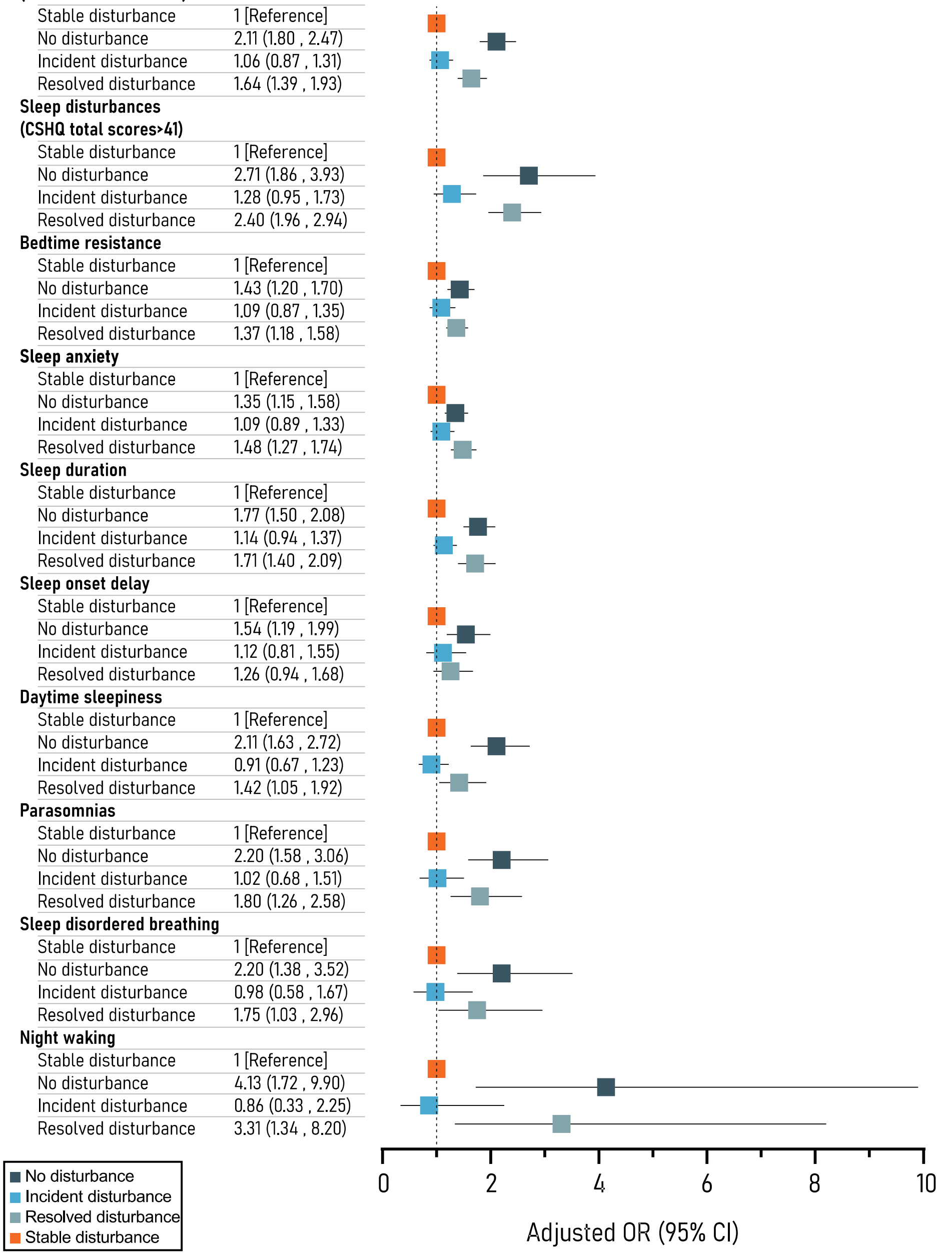

Comparisons are with children with stable sleep disturbances. All the *P* values are presented after Bonferroni multiple testing adjustments. AOR indicates adjusted odds ratio; CSHQ, Children's Sleep Habits Questionnaire.

© 2023 Deng Y et al. *JAMA Network Open*.

eReferences

1. Wang G, Takahashi M, Wu R, et al. Association between Sleep Disturbances and Emotional/Behavioral Problems in Chinese and Japanese Preschoolers. *Behav Sleep Med*. 2019:1-12.

2. Li SH, Jin XM, Shen XM, et al. Development and psychometric properties of the Chinese version of Children's Sleep Habits Questionnaire. *Zhonghua er ke za zhi = Chinese journal of pediatrics*. 2007;45(3):176-180.

3. Tinker EC, Garrison MM, Ward TM. Development of the Sleep Health in Preschoolers (SHIP) intervention: Integrating a theoretical framework for a family-centered intervention to promote healthy sleep. *Fam Syst Health*. 2020;38(4):406-417.

4. World Health Organization. Obesity and overweight, 2021. http://www.who.int/news-room/fact-sheets/detail/obesity-and-overweight (accessed Jul 6, 2023).

5. Guidelines on physical activity, sedentary behaviour and sleep for children under 5 years of age. *World Health Organization*. 2019.

6. Anme T, Tanaka E, T. W, Tomisaki E, Y. M, Tokutake K. Validity and Reliability of the Index of Child Care Environment (ICCE). *Public Health Frontier*. 2013;2(3):141-145.

7. Ip P, Tso W, Rao N, et al. Rasch validation of the Chinese parent-child interaction scale (CPCIS). *World J Pediatr*. 2018;14(3):238-246.

8. Staehr-Johansen K. Well-Being Measures in Primary Health Care – the DepCare

© 2023 Deng Y et al. *JAMA Network Open*.

Project. Geneva, World Health Organization: World Health Organization, Regional Office for Europe;1998.

9. White IR, Royston P, Wood AM. Multiple imputation using chained equations: Issues and guidance for practice. *Stat Med*. 2011;30(4):377-399.
