# Supplementary material for: Sleep Disturbances and Emotional and Behavioral Difficulties Among Preschool-Aged Children
Source: JAMA Netw Open. 2023 Dec 14;6(12):e2347623. doi: 10.1001/jamanetworkopen.2023.47623 (PMC10722331; doi:10.1001/jamanetworkopen.2023.47623)
Supplement: Supplement 2. — Data Sharing Statement [file jamanetwopen-e2347623-s002.pdf]

Data Sharing Statement

Deng. Sleep Disturbances and Emotional and Behavioral Difficulties Among Preschool-Aged Children. *JAMA Netw Open*. Published December 14, 2023.
doi:10.1001/jamanetworkopen.2023.47623

Data
Data available: No
